# Intravitreal Injection of Anti-VEGF Antibody Induces Glomerular Endothelial Cells Injury

**DOI:** 10.1155/2019/2919080

**Published:** 2019-12-21

**Authors:** F. Touzani, C. Geers, A. Pozdzik

**Affiliations:** ^1^Department of Nephrology and Dialysis, Centre Hospitalier Universitaire, Hospital Brugmann, Brussels, Belgium; ^2^Faculty of Medicine, Université Libre de Bruxelles (ULB), Brussels, Belgium; ^3^Department of Pathology, Centre Hospitalier Universitaire, Hospital Brugmann, Brussels, Belgium

## Abstract

**Introduction:**

Antiangiogenic agents that inhibit vascular endothelial growth factor have emerged as important tools in cancer therapy and ocular diseases. Their systemic use can induce renal limited microangiopathy. Local use of anti-VEGF agent is supposed to be safe. We report here a unique case of early endothelial cells injury induced by intravitreal injection of bevacizumab.

**Case Presentation:**

A 72-year-old man was addressed for acute kidney injury with proteinuria. He was under treatment with intravitreal injections of bevacizumab for glaucoma. Kidney biopsy was performed and electron microscopy showed signs of early stages of glomerular microangiopathy. Bevacizumab was discontinued resulting in the improvement of renal function and albuminuria.

**Discussion:**

Bevacizumab, a humanized monoclonal antibody to VEGF is an approved therapy for metastatic cancer. Systemic adverse events including thrombotic microangiopathy have been mainly reported after its systemic injection. Podocytes produce VEGF that interacts with endothelial cells VEGF receptor-2 maintaining glomerular basement membrane integrity. Bevacizumab induce the detachment of endothelial cells from glomerular basement membrane leading to the proteinuria and renal function decline. Intravitreal bevacizumab is generally supposed to be safe. However, glomerular injury with microangiopathy features, even after intravitreal injection is possible.

**Conclusion:**

We report the electron microscopy evidence that intravitreal injection of anti-VEGF induces glomerular endothelial cells injury. Nephrologists and ophthalmologists should be aware of this complication.

## 1. Introduction

Antiangiogenic agents that inhibit vascular endothelial growth factor (VEGF) have emerged as important tools to treat metastatic cancer and various ocular diseases [1, 2]. Their systemic use can induce nephrotoxicity, mainly glomerular injury characterized by minimal change disease, or most frequently features of renal limited microangiopathy, leading to proteinuria, nephrotic syndrome, and hypertension [3, 4]. This anti-VEGF induced renal microangiopathy is rarely associated to the classical hematologic abnormalities found in acute thrombotic microangiopathy (TMA), as it occurs among other things in typical, atypical haemolytic uremic syndrome, or malignant hypertension [4]. Intravitreal injection of anti-VEGF agent is supposed to be safe. However, systemic absorption may occur. We report here an exceptional case of kidney injury related to glomerular microangiopathy after intravitreal injection of bevacizumab, an anti-VEGF humanized antibody.

## 2. Case Presentation

**Figure 1:**
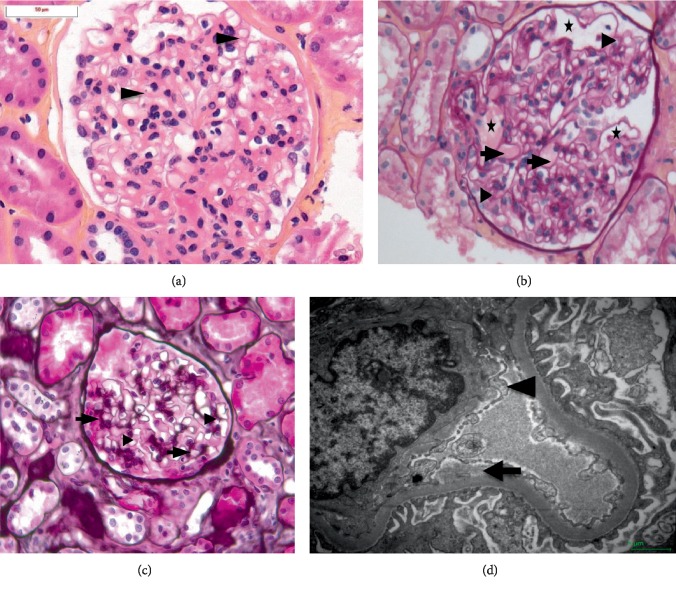
(a) Hematoxylin eosin stained sections show minimal thickening of capillary wall (arrowheads). Arterioles were normal (magnification ×400). (b) PAS stained slide showing a hyaline pseudothrombi (arrows) and segmental microaneurysms filled with pale material (stars). Double contours are visible (arrowheads) (magnification ×400). (c) Jones silver staining showing thickening of the capillary wall (arrows) and double contours (arrowheads). (d) Electron microscopy show focal loss of fenestrations of endothelial cells (arrowheads) and subendothelial space expansion by electron-lucent material (arrow) (magnification ×7000).

A 72-year-old man was addressed to the nephrology department for acute kidney injury with increased creatininemia at 2.2 mg/dL (*N*: 0.72–1.17) corresponding to an estimated glomerular filtration rate (eGFR) of 30 mL/min/1.73 m^2^ according to the CKD-EPI formula. He was asymptomatic. He had no personal or family history of kidney disease. He did not travel, had no allergies, and had no known contact with ill patients. He was not taking any nonsteroidal anti-inflammatory drugs. His medical history comprised of pleural tuberculosis treated 20 years before, well controlled hypertension under treatment with calcium antagonists and glaucoma treated by monthly intravitreal injections of bevacizumab in the past 6 months. Physical examination and blood pressure were normal. There was no swelling. Laboratory investigations did not show any sign of TMA, with normal haemoglobin and platelet count. Immunological testing was positive only for antiphospholipid antibodies. Infectious serology was negative. The urinalysis showed no haematuria but increase of albuminuria from 2.9 to 226 mg/g of creatinine (*N*: <30). Urine cultures were negative. Kidney ultrasound was normal. A renal biopsy performed two weeks after showed ten glomeruli. Two of them were obsolescent glomeruli with complete glomerulosclerosis. The other glomeruli demonstrated thickening of capillary wall (Figure 1(a)). There were segmental glomerular capillary microaneurysms, filled with pale material, and segmental hyaline thickening of the glomerular basement membrane (Figure 1(b)). Silver staining showed some double contours (Figure 1(c)). Mild focal interstitial fibrosis and tubular atrophy with a mononuclear cells infiltrate were seen. Immunofluorescence for IgA, IgG, IgM, C1q, kappa, and lambda was negative. However, C3 immunofluorescence showed a sparse endothelial positivity in arterioles (not shown). On electron microscopy, the endothelial cells showed irregularities, and focal loss of fenestrations. Subendothelial space expansion by electron-lucent material was visible (Figure 1(d)). This was consistent with signs of endothelial injury. Intravitreal injections of anti-VEGF were suspended. Four months after withdrawal, serum creatinine was of 1.20 mg/dL, and albuminuria normalized, supporting our hypothesis of intravitreal anti-VEGF induced renal microangiopathy. One year after bevacizumab was stopped, serum creatinine was of 0.95 mg/dL, and urinary albumin-creatinine ratio was of 3.3 mg/g. The patient did not present any thromboembolic event.

## 3. Discussion

Bevacizumab is a humanized monoclonal antibody to VEGF approved for the treatment of metastatic cancer, mainly colorectal, breast, and renal cell carcinoma [5]. By preventing the binding of VEGF-A to the endothelial cell surface receptors, it inhibits neovascularization, and then tumour growth [6]. The use of intravitreal bevacizumab has been successful in various ocular diseases with neovascularization, including exudative age-related macular degeneration (AMD), retinal neovascularization in proliferative diabetic retinopathy, or neovascular glaucoma as in our case [7].

Systemic adverse events are reported with the intravenous use of anti-VEGF therapy, including kidney injury with hypertension, proteinuria (even nephrotic syndrome), and features of TMA [8–11]. However, intravitreal anti-VEGF antibody injection is generally supposed to be safe. Within 1.173 patients treated with intravitreal bevacizumab and followed during one year, systemic side effects mainly hypertension, and cerebrovascular disease have been reported in 1.5% of cases. The most frequent complications were local, dominated by subconjunctival haemorrhage observed in 838 patients. No glomerular disease has been reported [12].

Kidney toxicity after intravitreal use of anti-VEGF is exceptional. This complication was first reported in a 77-year-old patient treated by intravitreal injections of ranibizumab (a Fab fragment of the bevacizumab humanized monoclonal antibody) for AMD. He presented nonimmune haemolytic anaemia associated with acute kidney injury and impure nephrotic syndrome. Kidney biopsy showed glomerular basement membranes duplications, endothelial swelling, and arteriolar thrombi compatible with TMA diagnosis. Electron microscopy study was not available. After discontinuation of ranibizumab injections, kidney function normalized [13]. Cheungpasitporn et al. described a 67-year-old man with polycystic kidney disease who underwent kidney transplantation and was treated by intravitreal injections of bevacizumab followed by aflibercept (another anti-VEGF-therapy) for AMD. He developed glomerular proteinuria that worsened during the follow-up. One year after transplantation, kidney allograft biopsy showed phospholipase A 2 receptor—negative membranous nephropathy but no TMA. The second case was a 52-year-old patient who underwent kidney transplantation for tuberous sclerosis. Sixteen years after transplantation, kidney biopsy was performed after progressive increase in proteinuria, and revealed acute, and chronic antibody-mediated rejection with glomerular thrombi. The patient had been started on intravitreal anti-VEGF therapy for AMD six months before. In these two cases, the temporal link of local anti-VEGF injections and allograft dysfunction has only been suggested but not demonstrated [14].

Our patient had positive antiphospholipid antibodies. He might present antiphospholipid antibody syndrome, a multisystem disorder that can induce TMA. We considered this diagnostic less likely as our patient improved his renal function and albuminuria after withdrawal of bevacizumab. Furthermore, glomerular anti-VEGF therapy induced microangiopathy display a histologic pattern that differs from other causes of TMA lesions, among other things the absence of fibrin thrombi, platelet thrombi, or fragmented erythrocytes [15]. Instead, segmental glomerular capillary microaneurysms filled with pale material are usually found as in our case. After local injection, bevacizumab has been detected in a very low serum concentration as compared to intraocular concentration (0.413 *µ*g/mL *vs* 406.25 *µ*g/mL) in experimental rabbit model [16]. In glomeruli, podocytes produce VEGF that binds to VEGF receptor-2 expressed on endothelial cells. This interaction is necessary to the normal function of the glomerular filtration barrier and for recovery of renal microvascular injury [17]. Ultrastructure study in a murine model of doxycycline induced VEGF deletion in podocytes showed swelling of the endothelial cells. This indicate the crucial role of VEGF in maintaining glomerular endothelium integrity [10]. The endothelial cells injury in our patient after intravitreal bevacizumab injections, indicate that even very low systemic concentrations of anti-VEGF could significantly impair interaction between VEGF derived from podocytes, and VEGF receptor on the endothelial cells.

As far as we know, we report for the first time the electron microscopy evidence that intravitreal injection of anti-VEGF induces glomerular endothelial cells injury. As the use of anti-VEGF therapy is increasing, particularly in ocular diseases with local injections, ophthalmologists, and nephrologists should be aware of this complication. A regular monitoring of renal function and proteinuria after introducing anti-VEGF antibodies is advised.
